# Cervical Paraspinal Chordoma: A Literature Review with a Novel Case Report

**DOI:** 10.3390/jcm11144117

**Published:** 2022-07-15

**Authors:** Redwan Jabbar, Jakub Jankowski, Agnieszka Pawełczyk, Bartosz Szmyd, Julia Solek, Olaf Pierzak, Maciej Wojdyn, Maciej Radek

**Affiliations:** 1 Department of Neurosurgery, Spine and Peripheral Nerve Surgery, Medical University of Lodz, 90-549 Lodz, Poland; rredwanbakal@gmail.com (R.J.); jjankowski.med@gmail.com (J.J.); agnieszka.pawelczyk@umed.lodz.pl (A.P.); bartoszmyd@gmail.com (B.S.); olafpierzak@gmail.com (O.P.); maciej.wojdyn@umed.lodz.pl (M.W.); 2 Department of Pathology, Chair of Oncology, Medical University of Lodz, 92-213 Lodz, Poland; julia.solek@umed.lodz.pl

**Keywords:** chordoma, chordoma untypical manifestation, primary bone tumors, surgical treatment, molecular targeted therapy

## Abstract

Chordomas are rare malignant neoplasms, accounting for 1–4% of all primary bone tumors. Most spinal chordomas occur in the sacrococcygeal region and the base of the skull; however, 6% of chordomas are observed in the cervical spine. In these cases, the lesion is mainly located in the midline. These tumors slowly grow before becoming symptomatic and encase the surrounding vascular and nerve structures. Patients with advanced chordoma have a poor prognosis due to local recurrence with infiltration and destruction of adjacent bone and tissues. Systemic chemotherapy options have not been fully effective in these tumors, especially for recurrent chordomas. Thus, new combinations of currently available targeted molecular and biological therapies with radiotherapy have been proposed as potential treatment modalities. Here, the present paper describes the case of a 41-year-old male with a C2–C4 chordoma located paravertebrally, who underwent surgical resection with a debulking procedure for a cervical chordoma. Computed tomography angiography revealed a paraspinal mass with bone remodeling and the MRI showed a paravertebral mass penetrating to the spinal canal with a widening of the intervertebral C2–C3 foramen. Initially, the tumor was diagnosed as schwannoma based on its localization and imaging features; however, the histopathology specimen confirmed the diagnosis of chordoma. This case study highlights the effectivity of radical surgical resection as a mainstay treatment for chordomas, discusses neuroimaging, diagnosis, and the use of currently available targeted therapies and forthcoming treatment strategies, as alternative treatment options for chordoma.

## 1. Introduction

Chordomas are rare malignant neoplasms with an estimated incidence of less than 0.1 per 100,000; this prevalence peaks in men around late middle age [1,2]. They account for 1–4% of all primary bone tumors. Most occur in the sacrococcygeal region and around the base of the skull. Chordoma of the cervical spine is observed only in 6% of all chordomas, and in these cases, the lesion is mainly located in the midline. These tumors slowly grow in size before becoming symptomatic and encase surrounding vascular and nerve structures. Patients with advanced chordoma have a poor prognosis due to local recurrence with infiltration and destruction of adjacent bone and tissues. As systemic chemotherapy options have been found to be not fully effective, molecular targeted therapies, especially tyrosine kinase inhibitors, have been proposed [3]. However, despite significant advances in therapeutic options, radical resection with subsequent postoperative radiotherapy remains the mainstay of care [1,4,5,6].

The current paper presents a case study of massive cervical (C2–C4) chordoma, located paravertebrally, which resulted in an initial diagnosis of schwannoma rather than chordoma. It discusses the features revealed by magnetic resonance imaging (MRI) and computed tomography angiography (CTA), the diagnosis, and currently available and upcoming treatment modalities, including targeted therapies.

## 2. Case Report

A 41-year-old man was admitted to our hospital with a slow-growing, retropharyngeal paravertebral mass on the right side of his neck, as revealed by an ultrasound scan. On admission, he reported a long history of sore throat, dizziness, and difficulty in swallowing, but the symptoms were transient. On clinical examination, the tumor was palpable, firm, non-pulsatile, and not moveable at the right cervical region. This finding was confirmed with an MRI. The past medical history and routine laboratory diagnostics were unremarkable and no significant neurological deficits were disclosed.

**Figure 1:**
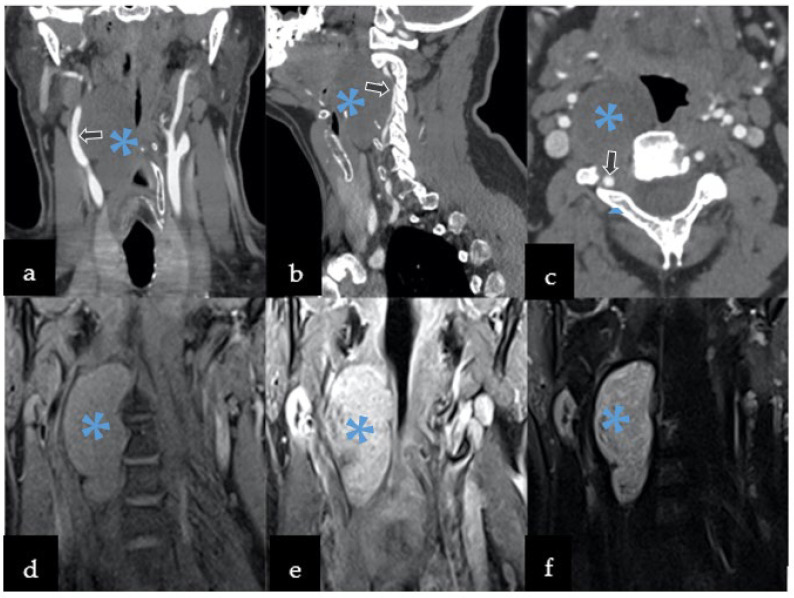
Preoperative imaging included both computed tomography angiography (CTA) and magnetic resonance imaging (MRI). CTA revealed hypodense well-defined mass with calcification displacing the vessels in the following planes: (**a**) coronal, (**b**) sagittal, and (**c**) axial. MRI was characterized by: (**d**) by low T1 signal, with (**e**) T1 contrast enhancement, and (**f**) heterogeneously hyperintense T2 signal. Legend: arrows—compression and displacement of the right carotid artery, *—lesion.

CTA revealed a 70 × 47 × 36 mm, well-defined paraspinal pathological mass with bone erosion and remodeling extending to the transverse processes and part of vertebra bodies, and with a widening of their intervertebral foramina on C2 to C4 (Figure 1a–c). It was performed to further rule out vessel infiltration and evaluate patency; the images identified compression and lateral displacement of the carotid artery. The right vertebral artery was also compressed and displaced posteriorly.

MRI showed the paravertebral mass penetrating to the spinal canal with a widening of the intervertebral C2–C3 foramen (Figure 1d–f). The tumor also compressed the larynx, pharynx, trachea, and soft parts of the neck, without any radiological signs of infiltration.

**Figure 2:**
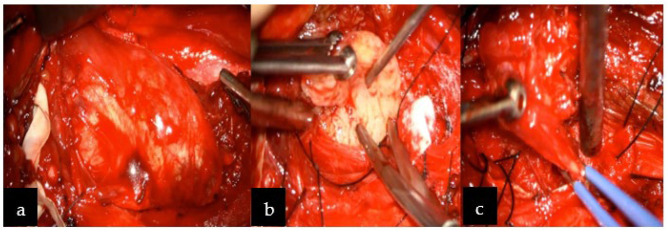
Intraoperative image revealing: (**a**) the paravertebral well-capsulated tumor on the right side at the C2–C4 level, (**b**) after debulking, and (**c**) complete excision was achieved. Below the tumor, vertebral artery was identified (not indicated in the image) and above the tumor, the hypoglossal nerve was visualized (not indicated in the figure).

Based on its localization, and neuroradiological and clinical features, the tumor was preliminarily diagnosed as a slow-growing lesion, such as peripheral nerve sheath tumor (PNST), more likely than meningioma (description Figure 1). Therefore, schwannoma diagnosis was more convincing than neurofibroma or malignant peripheral nerve sheath tumor (MPNST) due to homogenous enhancement on MRI without any infiltration to nearby structures and bone remodeling rather than destruction. The patient was qualified to surgery without prior preoperative biopsy, since performing biopsy in PNST carries risks of neurological deficit post-operatively, damaging adjusting vascular structures with consequent death [7,8]. Surgery was performed through an anterior transcervical approach procedure with a longitudinal incision from C2 to C6, along with the sternomastoid (Figure 2a–c). The cervical neurovascular bundle and vertebral artery were identified and dissected. Then, local structures were mobilized and pulled to the sides, exposing the tumor. Intraoperatively, a firm and well-encapsulated mass was seen along C2 to C4 under the microscope, which was carefully evacuated in fragments using an ultrasonic surgical aspirator. The lesion was fully dissected from the nerve roots and surrounding tissues, then removed. The patient’s postoperative course was uneventful, with discharge from the hospital after four days.

**Figure 3:**
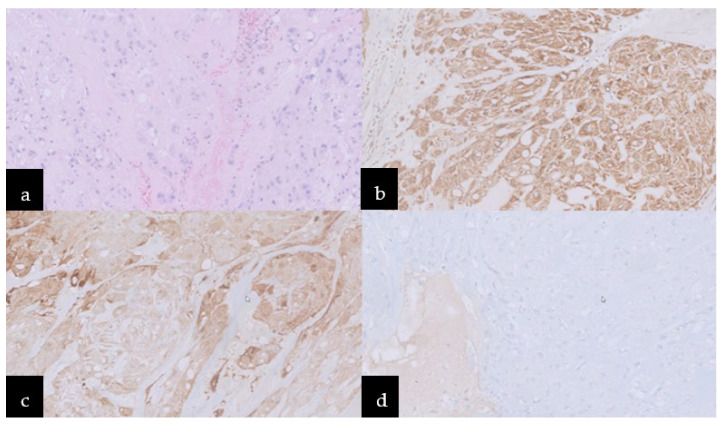
Microscopic images: (**a**) Hematoxylin–eosin stain, neoplastic cells with pale eosinophilic, cytoplasm vacuolated, arranged in cords separated by fibrovascular bands and myxoid matrix. Immunoreactivity for (**b**) AE1/AE3, (**c**) S100, and (**d**) negative one for GFAP.

Incidentally, the diagnosis of chordoma was established by histopathological features. The histopathological image revealed round neoplastic cells with a centrally located nucleus and pale eosinophilic, vacuolated cytoplasm, arranged in cords separated by fibrovascular bands and myxoid matrix. Nuclear pleomorphism was heterogeneous, but mitosis was rare (Ki67 < 1%). An immunohistochemical study revealed positive reactivity for EMA, AE1/AE3, S100, and negative for GFAP, which indicated notochordal origin and confirmed the final diagnosis of chordoma (Figure 3).

**Figure 4:**
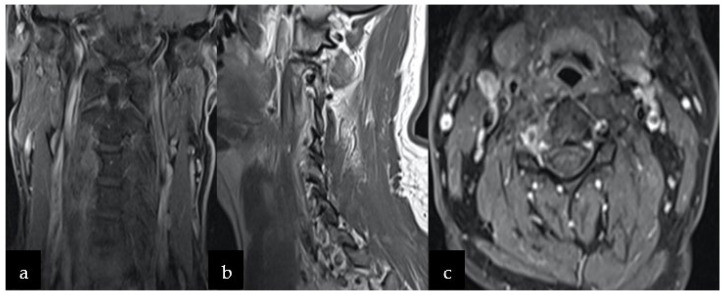
Follow-up MRI after 12 months showing no signs of recurrence on T-weighted 1 sequence with contrast in the following planes (**a**) coronal, (**b**) sagittal, and (**c**) axial.

Following consultation with a radiotherapist, oncologist, and neurosurgeon, the patient’s adjuvant radiotherapy was postponed and he was eventually placed under constant care. In the event of tumor recurrence, the consecutive follow-up MRI examinations after six and twelve months showed no tumor residue or recurrence (Figure 4a–c). Currently, the neurological state remains good without deficits, although the patient complains about transient dizziness.

## 3. Discussion

Chordomas are a family of primary bone tumors, which originate from undifferentiated embryonic notochord remnants present in the midline, extending throughout the skull base and the axial skeleton. This present case merits particular attention due to the unusual location of the tumor and the difficulties associated with the preoperative clinical and neuroradiological definitive diagnosis. In the current section, we decided to provide textbook-wise review juxtaposed with case discussion. We believe that this approach will be the most appropriate for clinicians, especially neurosurgeons and oncologists.

### 3.1. Epidemiological Data

Chordomas are the cause of 1–4% of all bone malignancies; they account for 0.5% of all primary CNS tumors and as much as 17% of spinal primary tumors. Population-based studies indicate the incidence rate of chordoma to be 0.08–0.1 per 100,000 in men and 0.06 per 100,000 in women [9,10]. The mean age at diagnosis is generally those in their 50s and 60s, with most cases being observed in men. Among girls, the most commonly observed histopathological type is the SMARCB1-deficient poorly differentiated chordoma [11].

Chordomas have the possibility of affecting all parts of the axial skeleton, with an almost equal incidence reported in the mobile spine (32.8%; especially on the midline), skull base (32%), and the sacrum (29.2%) [12]. The median overall survival depends on histopathological subtype and varies between 13 months and 48 months, depending on histopathological subtype. According to estimates, only 6% of all chordoma cases mainly affect the cervical spine, as chordomas can often be found in the cervical vertebrae, either a new primary tumor or a metastatic tumor [13]. Metastatic chordoma has been reported in about 9–30% of all cases, and this is commonly found in the lungs, liver, lymph nodes, bone, skin, brain, and cerebrospinal fluid [14]. Local progression also depends on histopathological type, varying between 46% in the case of classical chordoma and 65% in dedifferentiated chordoma. Survival ranges from 50 to 68% within 5 years and 28 to 40% within 10 years [15]. In the current case, the proper information about survival and the efficiency of the used therapeutic approach will be possible in further 5-year follow-up. Currently, the patient remains under the control of neurosurgeons and clinical oncologists.

### 3.2. Diagnostic Process in the COVID-19 Pandemic

The COVID-19 pandemic significantly impacted the possibility for conventional patient examination. Telemedicine emerged rapidly and has been an important tool in delivering virtual health—also in patients with brain and spine neoplasms. In addition, it has improved the access to specialists and supportive care remotely to centers specialized in cancer treatment for an initial diagnosis or second opinion. However, the failure to perform a conventional neurological examination, especially in cases with neurological deficits due to spine disease or tumor, in telemedicine might lead to misdiagnoses and legal implications. Additionally, telemedicine misses early signs, which are important to establish initial diagnoses and further assessment in neurological deficits, in cases which are difficult to distinguish in the variety of spinal diseases, such as myelopathy, peripheral nerve disease, and cauda equina syndrome. Moreover, adequate neuroimaging modalities are fundamental to establish a definitive diagnosis; however, it is difficult to obtain an early diagnosis in this kind of tumor. Telemedicine provides a unique opportunity for delivering convenient and safety health care to patients, but its permeant benefit in managing spine patients is still uncertain, as misdiagnosis or failure to indicate the baseline issue remotely, and lack of a standard legal framework, may create malpractice litigation and legal issues. Furthermore, several studies reported inverse correlation in the doctor–patient relationship with malpractice suits, which is hard to develop virtually. Further research is needed to overcome the barriers to implementing telemedicine into surgical care and also connecting surgical teams worldwide to provide better experience to patients [16,17].

### 3.3. Radiological Features

The majority of spinal chordomas occur on the midline and manifest themselves as a soft tissue mass with variable biological behaviors. There are four common types of chordoma: the classic and the chondroid forms, which are usually low-grade and locally aggressive tumors, the poorly differentiated form, and the dedifferentiated forms, which are more aggressive [18]. Typical radiological findings, which are characteristic of chordoma, comprise the presence of destructive and lytic bone lesions with sclerotic changes extending into the spinal canal [19,20].

The diagnosis commences with radiological imaging. An X-ray can uncover bone erosions with atypical calcium foci and lytic bone lesions. CT scans show hypodense masses with calcification and evaluate the extent of bone involvement and destructive patterns; these are accompanied by reactive sclerotic changes in 40–60% of cases [21,22].

An MRI surpasses other imaging modalities in diagnosing chordomas. It can delineate the extent of tumors and adjacent paravertebral structures and find where the tumors originate [21,22,23,24,25,26]. The multiplanar images produced by an MRI can be valuable in the preoperative planning of elective surgeries. Under gadolinium enhancement, chordomas can appear as heterogeneous hypointense masses on T1W images, depending on the existence of cystic degeneration, osteophytes, and calcifications. The lesion can also be hyperintense on T2W images, as in our present case [4,19,25].

Many spinal chordomas, as in the present case, manifest themselves as a massive paraspinal mass, with or without a lytic lesion of the vertebral bodies. These tumors can extend along with the vascular structures and nerve roots in proximity, and into the spinal canal, thus, enlarging the intervertebral foramina. Large tumors, such as schwannomas and neurofibromas, have very similar neuroradiological features, as noted in the aforementioned case, including a mass hypointense mass in T1, a hyperintense mass in T2-weighted images, and an enlarged intervertebral foramen, which cause regular vertebral scalloping [26,27,28]. These similarities can complicate preoperative diagnoses, emphasizing the importance of a multidisciplinary team for managing chordoma cases. In our case, CT and MRI images revealed the presence a paraspinal mass with well-defined margins, lytic changes in the C2–C3 vertebral bodies with sclerotic margins, and an enlargement of the right C2–C3 intervertebral foramen. These findings suggested a more slowly growing lesion, such as schwannoma or a neurofibroma. Consequently, chordomas with significant bone destruction may also be misdiagnosed as other spinal tumors, causing destructive bone changes, such as: metastatic carcinoma, chondrosarcoma, chordoid meningioma, myoepithelial tumor of bone [29], but also myeloma and lymphoma [21]. Although chondrosarcoma shares similar characteristic features with chordoma in MRIs, it affects the neural arch rather than the vertebral body [18]. The presence of intralesional calcifications is highly indictive of chordoma, but the multifocal changes within the spine localizations rule out a definitive diagnosis of chordoma [30]. Nevertheless, the imaging approach is not enough to make a proper diagnosis. It may be challenging, even in histopathological examination—therefore, some cases require brachyury staging, which helps to distinguish chordoma from chondrosarcoma, carcinoma, and chordoid meningioma [29]. Although the neuroimaging of the mass can be characteristic, histological examinations made by CT-guided biopsy tracks are crucial in formulating prognoses and attaining effective treatment plans [31,32].

The present case is unique for its atypical localization and neuroradiological features. Cervical chordomas are rare and mainly located on the midline with variable and extensive bone destructions and extension into the spinal canal. In our case, the tumor was located paravertebrally and invaded the adjusting vertebral bone, which is rare and can be misleading. Therefore, we believe that this case will be helpful in the diagnostic process and therapeutic planning for neurosurgeons and specialists involved in the management of similar cases of chordomas.

### 3.4. Chordoma Classification

Appropriate diagnosis and staging play significant roles in managing a suspected primary bone tumor in the spine. Recently, two staging systems have been used to classify spine tumors in treatment planning. The Enneking classification categorizes tumors from Stage I to Stage IIIB based on grade and extent. The Weinstein–Boriani–Biagini (WBB) classification allows some staging of spinal tumors and reveals the unique anatomical complexity of the spine [5].

Moreover, Stacchiotti and Sommer published the first guidelines for the diagnosis and treatment of chordoma in 2015 [33]. The ultimate goal of this classification and staging was to recommend the best approach for achieving tumor-free surgical margins, reliable tumor staging, and treatment guidance. The guidelines also demonstrate predictive and prognostic value regarding the risk of tumor recurrence risk and mortality in primary spinal tumors.

### 3.5. Therapeutic Options

Various chordoma treatment regimens and approaches have been attempted, including complete surgical resection, combined radiation, surgical excision, and radiotherapy. However, complete resection and determining widely negative surgical margins can often be challenging, given the proximity of the tumor to critical neurovascular structures, its slow growth patterns, the tendency to recur, and its resistance to traditional chemo- and radiotherapeutic modalities [34,35]. As such, to attain as radical a resection as possible while avoiding tumor spillage, the capsule should be left intact [36]. Aggressive surgical resection is typically used in conjunction with adjuvant radiotherapy, which causes an increase in the long-term remission phase and in the survival rate at 3–5 years survival to approximately 70% [4,31]. If total tumor removal is not able to treat the problem, palliative debulking followed by radiotherapy would be the next best option [34].

Surgical intervention and resection remain the standard treatment for chordomas and extent of the tumor resection is an important prognostic factor for patients with these tumors [37,38,39,40,41,42,43,44,45,46,47]. Chordomas have a tendency for seeding tumor cells around a surgical corridor, thus, *en bloc* resection without intraoperative capsule spillage and negative margins has been associated with a reduced recurrence rate [48,49]. These tumors occur mostly on the midline and tend to invade, adjusting critical nerve and vascular structure, limiting the ability to perform *en bloc* resection. The description of margins by the pathologist is important to distinguish between margins that contain tumorous cells or tissues and those that do not to perform *en bloc* resection precisely [6,18]. A multitude of patient- and tumor-specific factors, including tumor location, the neurovascular anatomy, as well as patient functional status, contribute to decision-making regarding surgical approach and extent of resection, with the aim of achieving as radical a resection as possible.

Moreover, biopsy should be performed prior to surgery for cases in which diagnosis of chordoma is suspected, which is important in overall therapeutic planning. However, this was not initially considered in our patient due to the presumptive diagnosis of PNST, as it is associated with a high risk of complications, such as neurological deficits and damaging surrounding vascular anatomy [7,8]. Recommendations for surgical margins vary on the basis of tumor grade or stage and presence of metastases; however, in some patients, radiotherapy or palliative interventions are recommended instead of radical resection [18]. CT-guided biopsy is favored for suspected chordomas because seeding of tumor cells might occur during open biopsy procedure; however, when the needle is used, the tract may be resected at the time of tumor resection. Biopsy and surgery should be carefully planned by an experienced neurosurgeon in coordination with other specialists so as to obtain a correct diagnosis and facilitate the management of patients with chordomas.

#### 3.5.1. Radiotherapeutic Approach

Because cervical chordoma is a rare entity, it is difficult to give recommendations regarding radiation treatment based on existing evidence. However, better outcomes have come through adjuvant radiotherapy [4]. The delivery of safe amounts of radiation is complicated by the proximity of chordomas to vital structures, in this case, the vertebral artery and the spinal cord [50]. A promising new method of resolving this problem is highly focused photon/proton beam therapy [1,50]; however, the timing of the radiation before and after surgery is more crucial than the type of particle delivered. Several studies reported that local control rates are improved when radiotherapy is delivered at primary resection compared to when the tumor recurs. Others have found pre-operative radiotherapy to demonstrate better outcomes than postoperative use. The proton-based radiation seems to be the gold standard. Its efficiency was confirmed, e.g., one retrospective study found focused image-guided proton-based radiation to be associated with better progression-free survival [51]. In another retrospective study, comparing 17 patients, some of whom only underwent surgery and others who underwent carbon ion therapy, the local recurrence-free survival rate at 5 years was 62.5% for the surgery group and 100% for the carbon ion radiotherapy group. In addition, the disease-specific survival rate at 5 years was 85.7% and 53.3%, respectively [52]. However, high-dose proton irradiation comes with a substantial cost and has limited accessibility. Comparative effects have been noted in gamma knife radiosurgery for chordoma, which offers a 62–76% tumor control rate and a 70% survival rate over a 5-year period [41,53,54]. Alternatively, in tumors with an epidural extension, brachytherapy can be used to deliver radiation to chordomas, with a lower risk of toxicity to the spinal cord [18].

Prospective trials have shown clear and definite evidence of local control by radiation alone or local control achieved by a combination of radiation and chemotherapy agents. Nonetheless, complete surgical resection will remain the main treatment. Most authors suggest that radical excision surgery followed by adjuvant radiotherapy should be the standard procedure performed for chordoma patients. On the other hand, Boriani et al. proposed that margin-free *en bloc* resection is sufficient [41].

Due to the rarity of the occurrence of cervical chordomas, no absolute therapeutic protocols or long-term evaluations have been reported, with all articles solely on a case-by-case basis [19,32]. Accordingly, there are no established and standardized therapies apart from surgery. Correspondent to chordoma’s tendency to recur locally, in particular, after subtotal resection, it has a limited response to radiotherapy and chemotherapy. Thus, the focus has shifted towards investigating genetic and molecular targeted therapy as a potential alternative/complementary therapeutic option.

Considering the rare nature of cervical chordomas, evidence-based recommendations regarding the management of cervical chordoma are difficult to attain; however, adjuvant proton beam and radiotherapy have been shown to improve the long-term remission rate and outcome [55,56]. Nevertheless, the decision of additional treatment should be made in the team of radiotherapists, oncologists, and neurosurgeons individually for each patient. In our case, there was no macroscopic residue and post-operative MRI did not show tumor recurrence. Following consultation with the radiotherapist, oncologist, and neurosurgeon, the patient’s adjuvant therapy was postponed and he was eventually placed under constant observation to monitor tumor recurrence and functional status.

#### 3.5.2. Current Research and Targeted Therapy

In addition to the treatment modalities mentioned above, other therapeutic options are being researched by Tamborini and Weinberger, such as targeting tyrosine kinase receptors, epidermal growth factor (EGFR), or platelet-derived growth factor receptors (PDGFR); all have been associated with chordoma development, either by activation or upregulation [57,58]. Recent case reports, retrospective case series, and some phase II clinical trials indicate that receptor inhibitors, such as imatinib, sorafenib, gefitinib, erlotinib, lapatinib, and sirolimus may also be effective treatments.

Imatinib is one of the most extensively studied receptor (PDGFR, KIT) inhibitors regarding the treatment of chordomas and brought about positive results in a phase II study that involved 50 patients. As such, 1 patient (2%) had a partial response, 11 patients had a lower response, 35 patients (70%) remained with stable disease, and 64% of the patients benefitted clinically [58]. Another retrospective trial showed the efficacy of imatinib when implemented together with sirolimus in 10 patients with advanced chordoma: one patient had a partial response, seven had stable disease, and one patient had progressive disease [59,60]. Hindi et al. confirmed the efficacy of imatinib, with 34 (74%) patients having stable disease after treatment [61]. In a phase II trial, Sorafenib treatment yielded a nine-month median progression-free survival rate of 73% and an 86.5% overall survival rate in a group of 27 patients with advanced and metastatic chordoma [62]. In a prospective phase II study examining the effectiveness of lapatinib among 18 patients with advanced and progressive EGFR-positive chordoma, it was found that only 33% (six patients) had a partial response and 38.9% (seven patients) had stable disease [62].

Other clinical trials are being conducted to investigate how afatinib, another EGFR inhibitor, works against chordomas. A previous comparison suggested that afatinib is a more promising agent then other EGFR inhibitors, yielding better results than the others. The cell lines that are most sensitive to afatinib activity lacked serine/threonine kinase 33 (STK 33) mRNA/protein levels; this suggests that STK 33 may be used as a potential biomarker for predicting the response to afatinib by the tumor. Of the tested inhibitors, lapatinib was found to be the least effective [1,63].

Other treatment options and inhibitors, which may be utilized, include: Cyclin D-dependent kinases (CDK4 and CDK6) inhibitors, Palbociclib, immune checkpoint inhibitors like Avelumab, and Nivolumab [64]. Most chordomas are observed as sporadic lesions. In other cases, they can be associated with TBXT gene duplication or even tuberous sclerosis, caused by germline loss-of-function mutations in the tumor suppressor gene (*TSC1* or *TSC2*) [65]. Studies have shown that aberration in genes play a fundamental role in tumorigenesis. Brachyury, as a transcription factor encoded by the TBXT gene, is associated with notochord development and maintenance [66]. TBXT gene duplications have been observed in family members and provided evidence for possible genetic predisposition to the development of chordomas [14,67]. As such, the pharmacological inhibition of H3K27-demethylases may represent a novel therapy that alters gene networks to survive tumors [68].

Immunotherapy is a promising treatment regimen for cancer since it shows promising results in managing a variety of malignancies. The efficacy of combining imatinib with everolimus is currently under investigation in adult patients who have INll-negative tumors, as is the use of oncolytic bacteria, tazemetostat, and nivolumab, with or without stereotactic radiation therapy [69].

Another promising area of research is the use of irradiated natural killer (NK) cells with cetuximab, since exposure to radiation increases antibody-dependent cetuximab cytotoxicity through overexpression of CD16 alleles on NK cells [70]. Chimeric antibody receptor therapy inhibits chondroitin sulfate proteoglycan-4, a cell surface molecule whose presence correlates with poor prognosis and metastasis [71,72]. Recent trials have examined the combined use of GI-6301 vaccines and radiotherapy. The radiation promotes infiltration by inflammatory cells and increases the expression of major histocompatibility complex (MHC) peptides, thus, raising the amount of T cell activity against cancer cells (NCT02383498) [73]. These treatment modalities are laying down the foundation for more effective therapeutical options in the future.

Our case underlines the effectiveness of radical surgical resection as a mainstay treatment for chordoma and discusses the use of currently available biological agents as well. Disease control lasted for 12 months and a follow-up cervical MRI revealed no further progression and recurrence.

## 4. Conclusions

As chordomas remain a rare entity, planning treatments and standardizing systemic therapy constitutes a challenge in atypical cases. Here, we presented the case of a C2–C4 chordoma located paravertebrally and resembling PNST in the preoperative examination. In addition, as a limited number of randomized trials have been carried out, chordoma has only a few systemic treatment options, most of which are of limited benefit. Targeted therapy has been found to be a potential alternative treatment for chordoma, especially in cases with several relapses. Although the treatment options for recurrent chordoma are slowly growing, the condition is still burdened by significant morbidity and mortality. Hence, there is a need for further research into new therapeutic strategies. Currently, total marginal resection with the possible addition of adjuvant radiotherapy remains an effective therapeutic option. In such cases, detailed preoperative planning, by a multidisciplinary team, is crucial to achieve the best possible patient outcome.

## Data Availability

Not applicable.
